# Towards Context‐Specific EHDI Services: Understanding Caregivers' Priorities and Preferences in South Africa Through a Conjoint Analysis

**DOI:** 10.1111/cch.70090

**Published:** 2025-04-28

**Authors:** Katijah Khoza‐Shangase, Ntsako P. Maluleke

**Affiliations:** ^1^ Department of Audiology, School of Human and Community Development University of the Witwatersrand Johannesburg South Africa

**Keywords:** conjoint analysis, discrete choice experiment, early intervention, EHDI, hearing impairment, South Africa

## Abstract

**Background:**

Early Hearing Detection and Intervention (EHDI) programmes are critical for addressing hearing impairment in children by ensuring timely diagnosis and intervention. However, systemic barriers such as linguistic diversity, financial constraints and geographic accessibility challenges hinder equitable access to EHDI services in South Africa. This study explores caregivers' preferences for key attributes of EHDI services to inform the development of context‐specific and family‐centred programmes.

**Methods:**

A cross‐sectional survey utilizing conjoint analysis was conducted with 31 caregivers of children with hearing impairment enrolled in early intervention preschools in Gauteng, South Africa. Participants evaluated five key attributes of EHDI services: language of service provision, location of diagnostic evaluations, mode of early intervention delivery, integration of support services and cost reduction strategies. Data were analysed using the conditional logit model to determine attribute preferences.

**Results:**

Caregivers preferred services provided in their home language, diagnostic evaluations conducted at the nearest healthcare facility, home‐based early intervention sessions, regular integration of support services and reductions in the cost of EHDI services. Reluctance to adopt telehealth was also noted, highlighting concerns about technological literacy, internet access and trust in virtual care delivery. These preferences emphasize the need for accessible, linguistically congruent and affordable EHDI services tailored to the South African context.

**Conclusions:**

This study provides valuable insights into caregivers' preferences for EHDI services, offering actionable recommendations to address systemic barriers such as designing EHDI services that address systemic barriers to ensure equity in healthcare access. Policymakers and stakeholders should prioritize linguistically diverse service delivery, improve healthcare facility accessibility, incorporate continuous informational counselling and reduce financial burdens to ensure equitable and family‐centred EHDI programmes. Future research should explore preferences among a more geographically and socio‐economically diverse population to further refine these recommendations. While grounded in South Africa, the results provide insights applicable to other low‐ and middle‐income countries (LMICs) with similar challenges.

## 1 Introduction

Early Hearing Detection and Intervention (EHDI) is recognized as the standard of care for newborns and infants presenting with a hearing impairment, and encompasses the earliest possible identification, diagnosis and provision of intervention for newborns and infants with hearing impairment, to ensure that these children communicate effectively and develop to their maximum potential (Health Professions Council of South Africa‐HPCSA 2018; Naidoo and Khan 2022). A review of published literature on EHDI within the South African context highlights the country's considerable efforts towards implementation of these programmes (Hussein et al. 2018; Kanji 2016; Naidoo and Khan 2022; Maluleke et al. 2024). However, while there has been progress in advancing EHDI initiatives, significant gaps remain in ensuring these services are both accessible and contextually relevant to the diverse population within South Africa. Despite the documented progress, EHDI programmes within this context are still limited, resulting in late diagnosis and intervention for children with hearing impairment (Ehlert and Coetzer 2020; Hussein et al. 2018; Khoza‐Shangase 2019; Störbeck and Young 2016; Swanepoel and Clark 2019; Maluleke et al. 2024; Petrocchi‐Bartal et al. 2025).

South Africa's unique sociolinguistic and socio‐economic landscape presents specific challenges for the equitable delivery of EHDI services. The country's 11 official languages, combined with high levels of income inequality, necessitate a tailored approach to healthcare delivery that can address these barriers. Systemic inequalities within the healthcare system often result in delayed service delivery, further compounding the difficulties experienced by caregivers of children with hearing impairment. These issues underscore the importance of adopting a family‐centred approach to EHDI, one that acknowledges and integrates the preferences and priorities of caregivers. Given South Africa's diverse linguistic and socio‐economic landscape, it is imperative to develop EHDI services that are culturally and contextually aligned with caregivers' needs (Khoza‐Shangase 2025).

Moreover, challenges with accessing EHDI services due to capacity versus demand challenges; differences between the care in public and private healthcare sectors; as well as language and cultural incongruence between the general public and the largely white, female, English‐ or Afrikaans‐speaking staff complement have also been reported (Hussein et al. 2018; Khan et al. 2018; Khoza‐Shangase 2019; Khoza‐Shangase and Kanji 2021; Pillay et al. 2020). Language disparities, in particular, have been identified as critical obstacles to effective healthcare access, as families often encounter linguistic incongruence between themselves and healthcare providers (Khoza‐Shangase and Kanji 2021). Furthermore, rural–urban disparities exacerbate issues of accessibility, with rural areas often facing limited access to diagnostic and intervention services (Khoza‐Shangase 2021). Furthermore, despite a waiver of user fees being implemented at public healthcare facilities; EHDI services are not affordable to the general public due to the high costs associated with these services, amplification devices and accessories within the private sector, as well as transport costs to public healthcare facilities (Maluleke et al. 2024; Khoza‐Shangase 2019; Khoza‐Shangase 2021).

These structural and systemic barriers highlight the urgent need for research that delves into caregivers' lived experiences and preferences to inform the design of responsive EHDI programmes. For instance, caregivers' preferences regarding language of service delivery, proximity of healthcare facilities and affordability are critical factors that must be considered in service planning and implementation. These challenges underscore the need for context‐specific research within the South African context to guide best practice that is relevant and responsive to these contextual realities (Khoza‐Shangase 2021; Khoza‐Shangase et al. 2017; Kanji and Khoza‐Shangase 2021). Feasibility of, approach to, barriers and facilitators of, as well as the models of care of EHDI programmes have been investigated and documented within this context; however, investigations of caregivers' preferences for characteristics associated with EHDI programmes are lacking within this context. Preference in healthcare refers to the specific activity, treatment and provider conditions that patients desire for their healthcare experience (Swift et al. 2021). Considering patient preference when making healthcare decisions is increasingly considered an essential element of evidence‐based practice (Dirksen et al. 2013). Within the South African context, where systemic barriers to healthcare persist, understanding caregiver preferences is not merely a matter of improving service delivery but a critical step in advancing health equity and ensuring that interventions are inclusive and accessible. When intervention is considered to be preferable, patients are more likely to adhere to treatment recommendations, which may impact on the overall effectiveness of the intervention and improved outcomes for patients (Sekhon et al. 2017; Mtimkulu et al. 2023).

Thus, the current study aimed to explore caregivers' preferences for characteristics associated with EHDI services for caregivers of children with hearing impairment. This study focuses on caregivers as important co‐drivers of any early intervention programme. By investigating caregivers' preferences through conjoint analysis, this study seeks to provide actionable insights that can inform the development of culturally and contextually aligned EHDI programmes in South Africa. This study forms part of a larger research project titled ‘Family‐Centered EHDI: Caregivers' experiences and evaluation of the process and practices in the South African context’, describing the first steps in formulating a framework for FC‐EHDI for children with hearing impairment within the South African context. Globally, disparities in access to EHDI services are not unique to South Africa. Rural communities in high‐income countries and resource‐constrained settings in low‐ and middle‐income countries (LMICs) face similar challenges, including linguistic barriers, affordability issues and geographic accessibility. Lessons from the South African context can inform strategies to enhance EHDI programmes globally, particularly in addressing systemic barriers to equity in healthcare.

## 2 Materials and Methods

### 2.1 Study Design

Results of the current study are based on an online survey, questionnaire‐based conjoint analysis that formed part of phase 2 of the main study. Conjoint analysis was chosen for this study as it allows for a nuanced understanding of caregivers' preferences by evaluating trade‐offs among multiple service attributes. This method is particularly suited to the South African context, where socio‐economic and linguistic diversity necessitate the development of context‐specific EHDI services tailored to caregivers' priorities.

### 2.2 Participants

Non‐probability purposive sampling was used in this study. Thirty‐one of the forty (77.5%) caregivers recruited to participate in the study completed the conjoint analysis questionnaire. Caregivers who participated in the study met the following inclusion criteria:

- Caregiver of a child diagnosed with a mild to profound, bilateral, or unilateral hearing impairment
- Caregiver of a child who was enrolled in an EI preschool centre in Gauteng, South Africa, between 2010 and 2020

The sample size was determined based on the study's methodological approach and logistical constraints, including the availability of caregivers within early intervention preschools in Gauteng. Given the specificity of the population and the exploratory nature of the study, a smaller sample size was deemed appropriate to generate preliminary insights into caregiver preferences.

Gauteng was selected as the study site due to its representation of urban and peri‐urban populations, providing insight into the preferences of a diverse group of caregivers across various socio‐economic strata. However, the study acknowledges that further exploration in rural settings is required for broader generalizability. Caregivers were purposively sampled to ensure representation across key demographic factors, including ethnicity, language and socio‐economic background, reflecting the diversity of the South African population. Although this study focused on urban and peri‐urban caregivers in Gauteng, similar urban–rural disparities in healthcare access have been documented globally. Current findings provide a foundation for future research to explore caregiver preferences in other settings to better understand the universality of these barriers.

Table 1 summarizes participants' socio‐demographic information.

**TABLE 1:** Participants' socio‐demographic profile.

Heading	Subheading	Participants *N* (%)
Gender	Male	4 (12.9%)
	Female	27 (87.1%)
	Prefer not to answer	0
Age	35 years or younger	6 (19.4%)
	36–45 years	22 (71%)
	46–55 years	3 (9.7%)
	56 years and older	0
	Prefer not to answer	0
Ethnicity	Black	12 (38.7%)
	White	11 (35.5%)
	Coloured (mixed race)	5 (16%)
	Indian	2 (6.5%)
	Prefer not to answer	1 (3.2%)
Marital status	Single	6 (19.4%)
	Married	24 (77.4%)
	Divorced	1 (3.2%)
	Separated	0
	Widow	0
	Prefer not to answer	0
Highest qualification	Grade 11 or lower (Std 9)	2 (6.5%)
	Grade 12 (matric)	11 (35.5%)
	Postmatric diploma	8 (25.8%)
	Baccalaureate degree(s)	3 (9.7%)
	Postgraduate degree(s)	6 (19.4%)
	Prefer not to answer	1 (3.2%)
Employment status	Student	0
	Employed	22 (71%)
	Self‐employed	8 (25.8%)
	Prefer not to answer	0
	Unemployed	1 (3.2%)
Languages spoken	English	8 (26%)
	Bilingual with South African Sign Language	4 (13%)
	Sesotho	5 (16%)
	Setswana	2 (6.5%)
	Sepedi	2 (6.5%)
	Portuguese	2 (6.5%)
	Afrikaans	5 (16%)
	IsiZulu	3 (9.7%)

Source: Original.

Most of the participants were female (87.1%), between the ages of 36 and 45 years (71%), married (77.4%), with a Grade 12 (35.5%) highest level of qualification and employed (71%). Of the participants, 38.7% were Black, 35.5% were White, 16.1% were Coloured and 6.5% were Indian. They spoke a variety of languages including bilingualism, with SASL as a second home language. Most notable, only 26% spoke English as a home language.

### 2.3 Ethical Considerations

Prior to the commencement of this study, ethical clearance was obtained from the University of the Witwatersrand's Human Research Ethics Committee (Medical) (Protocol Number: H19/06/16). Furthermore, the work adhered to the Helsinki Declaration of 1975, as revised in 2013 (World Medical Association 2013).

### 2.4 Materials

Data were collected using a Google Forms conjoint analysis questionnaire. The questionnaire was in English and comprised of two sections, a demographic questionnaire section and a conjoint analysis section.

A conjoint analysis (or discrete choice experiment) is an evaluation approach that uses survey methods to elicit trade‐offs among attributes and attribute levels to determine respondents' preferences for alternative products (Eggers et al. 2021; Marshall et al. 2010). It is a method of eliciting stated preferences from patients and provides an estimation of the relative importance people attribute to various components of care, measures how individuals are willing to trade between these characteristics and estimates the overall satisfaction they gain from various forms of health service provision (Ali and Ronaldson 2012; De la Cuesta et al. 2022; Eggers et al. 2021).

**FIGURE 1:**
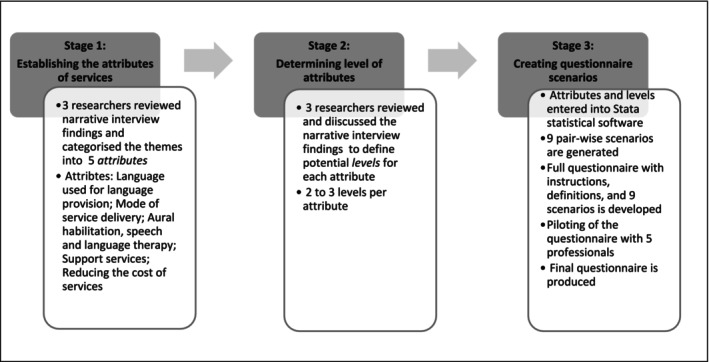
Description of conjoint analysis questionnaire development. *Source:* Original

The conjoint analysis questionnaire was developed according to the steps as outlined in Johnson et al. (2013). The development of a conjoint analysis questionnaire is made up of three stages, namely, establishing the attributes of services, determining level of attributes and creating questionnaire scenarios, as depicted in Figure 1.

#### 2.4.1 Stage 1: Establishing the Attributes of Services

This stage involved identifying the principal attributes and features or characteristics of the services. The narrative interview findings previously reported in Maluleke et al. (2023) informed the content of the questionnaire. A literature review was also conducted to supplement the identified attributes and levels. Five main attributes were included to ensure that the conjoint analysis survey instrument was manageable. The attributes and levels included in the conjoint analysis questionnaire were informed by extensive narrative interviews with caregivers, as well as a review of local literature on barriers to EHDI services in South Africa, such as linguistic incongruence, cost and geographic accessibility.

#### 2.4.2 Stage 2: Determining Level of Attributes

In this stage, the levels (options) of care were defined for each attribute. The level represents a choice that describes an attribute, and each attribute was defined by three levels, resulting in the creation of scenarios.

The final list of attributes and levels is provided in Table 2, below.

**TABLE 2:** Attributes and attribute levels.

Attributes	Attribute levels		
Language used for service provision	English	Home language	Any one of the South African languages.
Diagnostic evaluation	Child receives EHDI services and is referred to other HCWs (i.e., paediatrician, social worker and psychologist) of the caregiver's choice for all the other services that the child/and or caregiver need.	Child receives EHDI services at the nearest healthcare facility that offers all services (i.e., ENT and psychology) that the child/and or caregiver need.	Child receives all EHDI services (i.e., audiology, speech therapy, ENT and psychology) via telehealth. Child and caregiver might be required to go to the healthcare facility for some tests/procedures.
Aural habilitation, speech and language therapy	Regular direct therapy sessions (i.e., once a week) at the nearest healthcare facility.	Regular sessions (i.e., once a week) at home with the child, caregiver and HCW.	Regular sessions (i.e., once a week) at home with the child, and caregiver via telehealth. Direct therapy is then offered if there is a speech and/or language delay or no progress.
Support services	Caregivers' information sessions.	Regular part of services according to the child, caregiver and family's needs.	Caregivers seek information independently.
Reducing the cost of services	Decrease transport cost to healthcare facility.	Decrease cost of services, i.e., screening, evaluation, aural habilitation and speech and language therapy.	Decrease cost of amplification devices, FM systems, etc.

Source: Original.

The base levels for each attribute were selected based on a combination of current standard practices in EHDI services in South Africa, existing literature on caregiver preferences and expert consultations with audiologists and early intervention specialists. The base levels were defined as the most commonly available or widely accepted option within the South African healthcare context, serving as the reference category against which other attribute levels were compared. This approach ensured that the relative importance of different levels could be meaningfully interpreted within the constraints of real‐world service delivery.

#### 2.4.3 Stage 3: Creating Scenarios

Once the attributes and levels were determined, these were combined into were combined into pairwise choices, for each scenario. All the possible pairwise choices would render the questionnaire unmanageable, thus the scenarios were developed using a fractional factorial design in STATA. Nine scenarios with pairwise choices identified as ‘Service A’ and ‘Service B’, with the carrier phrase ‘Which service do you prefer?’ were included in the study. A fractional factorial design was employed to manage the complexity of the conjoint analysis while ensuring statistical efficiency. This approach allowed for the estimation of main effects without requiring an exhaustive number of choice sets.

Before distribution, the questionnaire was reviewed by the researchers for appropriateness and clarity. Subsequently, a final step of revisions and pilot testing of the questionnaire for clarity and ease of completion was conducted with five audiologists working with children with hearing impairment and two caregivers of children with hearing impairment. Minor revisions were conducted thereafter.

### 2.5 Data Collection Procedures

Participants were recruited to participate in this study telephonically. Once participants indicated an interest in participating in the study, an information letter and consent form were sent to them via email. Once informed consent was obtained, the questionnaire was sent via email or WhatsApp. The questionnaire was sent out as a Google form to ensure anonymity of participants' responses; thus, the main researcher sent reminders to all participants who had not confirmed completion of the questionnaire. Three reminders were sent to participants if they had not responded by the set date. The main researcher sent a reminder at the end of week one, and another one at the end of week three. The third reminder was sent at the end of week 5. To accommodate the linguistic diversity of participants, the information letters and consent forms were translated into multiple South African languages upon request. This ensured that participants fully understood the study's purpose and procedures. Recognizing potential disparities in access to technology, caregivers were given the option to complete the questionnaire via WhatsApp or email, ensuring inclusivity in participation.

### 2.6 Data Analysis

The conjoint analysis results were analysed using the conditional logit model, using STATA 17. The conditional logit model is the method mostly used in conjoint analysis and analyses preference data (Wang et al. 2019; Soekhai et al. 2019). The conditional logit model represents participants' choices as a function of the choice's characteristics; thus, enabling the researcher to predict participants preferences of attributes on condition of the attribute levels (Shi et al. 2018; Soekhai et al. 2019). The conditional logit model was selected as it allows for the prediction of preferences based on caregivers' trade‐offs between competing attributes. This approach is ideal for identifying the relative importance of attributes in a context where resource allocation and service delivery must be optimized for diverse populations. For this study, aggregate data of participants responses for each attribute were used during data analysis.

## 3 Results

The results highlight caregivers' preferences for EHDI service attributes within the South African healthcare context.

Tables 3 and 4 details the results from the conditional logit. Negative coefficients indicate that the attribute level is less preferred (reluctance) than the base level. The results will be discussed according to the attributes.

**TABLE 3:** Results of the conditional logit model highlighting caregivers' preferences for EHDI service attributes within South Africa's diverse healthcare landscape.

Attributes	Levels	Correlation coefficient (*r*)	*p* value	95% confidence interval
Language used for service provision	Any language	Base level		
	English	1.466	0.011*	0.37 to 2.85
	Home language	1.609	0.022*	0.21 to 2.721
Diagnostic evaluation	Child receives EHDI services and is referred to other HCWs of the caregiver's choice	Base level		
	Child receives EHDI services at the nearest healthcare facility	0.262	0.288	−0.577 to 1.152
	Child receives all EHDI services via telehealth	−0.223	0.105	−0.795 to 1.006
Aural habilitation, speech and language therapy	Regular sessions at home with the child, caregiver and HCW	Base level		
	Regular direct therapy sessions at the nearest healthcare facility.	−0.693	0.134	−1.600 to 0.214
	Regular sessions via telehealth	−0.336	0.416	−1.148 to 0.475
Support services	Caregivers seek information independently	Base level		
	Caregivers' information sessions.	−1.52e−16	1.000	−0.877 to 0.877
	Regular part of services	0.095	0.827	−0.761 to 0.951
Reducing the cost of services	Decrease cost of amplification devices	Base level		
	Decrease cost of services	0.262	0.533	−0.562 to 1.087
	Decrease transport cost to healthcare facility	−0.223	0.638	−1.153 to 0.707

Source: Original.

* *p* < 0.05.

**TABLE 4:** Ranking of caregiver preferences for EHDI attributes, reflecting socio‐economic and linguistic priorities in the South African context.

Attributes	Attribute levels (from most preferred to least preferred)		
Language used for service provision	Home language	English	Any language
Diagnostic evaluation	Child receives EHDI services at the nearest healthcare facility	Child receives EHDI services and is referred to other HCWs of the caregiver's choice	Child receives all EHDI services via telehealth
Aural habilitation, speech and language therapy	Regular sessions at home with the child, caregiver and HCW	Regular sessions via telehealth	Regular direct therapy sessions at the nearest healthcare facility.
Support services	Regular part of services	Caregivers seek information independently	Caregivers' information sessions.
Reducing the cost of services	Decrease cost of services	Decrease cost of amplification devices	Decrease transport cost to healthcare facility

Source: Original.

For the first attribute, ‘language used for service provision’, participants had a stronger preference for EHDI services to be provided in their home language (*r* = 1.609) than in any language (base level), if an interpreter was provided using English; and a strong preference for these services to be provided in English (*r* = 1.466) than the base level. The preference for home language services underscores the critical need for linguistically congruent healthcare delivery in South Africa. This finding aligns with national efforts to reduce linguistic barriers, particularly in communities where caregivers may not be fluent in English. It also highlights the need for training healthcare providers in multiple South African languages or incorporating interpreter services into EHDI programmes.

For the second attribute, ‘diagnostic evaluation’, participants had a weak preference for receiving EHDI services from their nearest clinic (*r* = 0.262) compared with receiving services from an HCP of their choice (base level). Furthermore, participants unpreferred receiving services via telehealth (*r* = −0.223) compared with receiving these services from an HCP of their choice. Caregivers' preference for services at the nearest healthcare facility reflects the significant impact of geographic accessibility on healthcare utilization, particularly in peri‐urban and rural areas. The observed reluctance towards telehealth may be attributed to limited digital literacy, inconsistent internet access, or concerns about the quality of remote care. This highlights an opportunity for healthcare policymakers to explore hybrid service models that combine telehealth with in‐person visits to mitigate these challenges.

For the third attribute, ‘aural habilitation, speech and language therapy’, participants unpreferred receiving aural habilitation and/or speech and language therapy services via telehealth (*r* = −0.336) and from their nearest HCF (*r* = −0.693) compared with receiving these services at home (base level). The strong preference for home‐based therapy sessions indicates caregivers' desire for family‐centred care that minimizes travel burdens and integrates intervention into the child's natural environment. This approach may enhance caregiver participation and adherence to therapy recommendations, ultimately improving developmental outcomes for children.

For the fourth attribute, ‘support services’, participants had a weak preference for receiving support services as a regular part of EHDI services (*r* = 0.095) compared with seeking information about support services independently. Furthermore, participants unpreferred attending caregiver information sessions (*r* = −1.52e−16) compared with seeking for information independently. Caregivers' preference for regular support services suggests a need for continuous informational counselling throughout the intervention process. This aligns with global evidence emphasizing the role of consistent support in reducing caregiver stress and improving adherence to treatment plans. In the South African context, this highlights the importance of equipping healthcare providers with culturally sensitive counselling skills and ensuring that support services are accessible to caregivers from diverse backgrounds.

For the fifth and last attribute, ‘reducing the cost of services’; participants had a weak preference for reducing the cost of EHDI services (*r* = 0.262) to make them more affordable compared with reducing the cost of amplification devices. Furthermore, participants unpreferred reducing transport costs (*r* = 0.223) compared with reducing the cost of amplification devices for these services to be more affordable. The preference for reducing the overall cost of EHDI services reflects the significant financial barriers faced by caregivers in South Africa, where income inequality and high unemployment rates exacerbate challenges in accessing care. This finding underscores the urgency of policy interventions aimed at subsidizing costs or integrating EHDI services into publicly funded healthcare packages.

Notably, results for ‘language used for service provision’ had a strong degree of associating (*r* > 0.7) and were statistically significant with *p* value less than 0.05. However, the correlation coefficients for the attribute levels for ‘mode of service delivery’, ‘aural habilitation, speech and language therapy’, ‘support services’ and ‘reducing the cost of services’ were less than 0.6 a indicating a low degree of association between the variables. Furthermore, the *p* values for these attribute levels were greater than 0.05 indicating that these results were not statistically significant. While the attributes ‘diagnostic evaluation’, ‘aural habilitation, speech and language therapy’, ‘support services’ and ‘reducing the cost of services’ showed weak correlation coefficients (*r* < 0.7) and *p* values > 0.05, these findings still provide valuable preliminary insights.

The use of a fractional factorial design, while statistically efficient, may have influenced the ability to detect significant differences in certain attributes. Specifically, the limited number of choice sets may not have fully captured all possible interactions between attributes, potentially contributing to the non‐significant findings observed in some variables. Future studies utilizing a full factorial design or alternative experimental approaches may further elucidate these relationships. Therefore, future research should aim to validate these results with a larger, more diverse sample to strengthen the evidence base.

## 4 Discussion

Traditionally, patients' involvement in decision‐making at both the microlevel of patient consultation and macrolevel of programme planning and development has been minimal (Ryan and Farrar 2000; Vahdat et al. 2014). To the best of our knowledge, the current study is the first study to explore caregivers' preferences for characteristics associated with EHDI services. Results from this study indicated that caregivers preferred that EHDI services be (1) conducted in their home language, (2) diagnostic evaluations be conducted at their nearest HCF, (3) early intervention services be provided at their home, (4) support service be provided as part of early intervention services and that (5) the cost of EHDI services be decreased.

Findings of caregivers' preference that EHDI services be provided in their home language resonates with findings reported by Khoza‐Shangase (2019) and Maluleke et al. (2023), where use of English during EHDI services was identified as a barrier to accessing these services—as has been reported in multicultural contexts such as Canada and India, where healthcare services often fail to accommodate the linguistic diversity of their populations. Furthermore, caregivers' preference of the use of English, second to use of their home language may be attributed to English being the prominent language in political, educational and social settings (Pascoe et al. 2018). Provision of EHDI services in a family's home language has been recommended as a way of mitigating this language barrier. Furthermore, using the language that the family can understand has been shown to improve the degree of accurate recall and has significant implications for follow‐up of treatment options, commitment and adherence to treatment recommendations and improved health outcomes (Khoza‐Shangase and Mophosho 2018; Watermeyer et al. 2017; Watson and McKinstry 2009). Global evidence on health indicates that patients who do not form part of the dominant language have worse health outcomes than patients who form part of the dominant language (Flood and Rohloff 2018). In South Africa, implementing linguistically congruent services can help bridge gaps in healthcare equity, particularly in multilingual and underserved communities, where effective communication is paramount for fostering trust and adherence. Thus, this finding raises significant implications for robust efforts to embrace linguistically congruent EHDI services within the South African context.

The second finding of the study revealed caregiver's preference of receiving diagnostic evaluations from their nearest HCF, as opposed to receiving these services from a caregiver of their choice or via telehealth. Distance to HCFs has also been identified as a barrier to accessing healthcare in South Africa (McLaren et al. 2014). In Kanji and Khoza‐Shangase's (2018) study, distance from HCFs negatively influenced follow‐up return rates in newborn screening programmes; while in Maluleke et al.'s (2023) study, participants expressed frustration with having to travel long distances in sometimes hard‐to‐reach areas in order access EHDI services. Telehealth has been proposed as a model for improving healthcare access in resource‐constrained contexts such as South Africa (Swanepoel and Hall 2010; Sebothoma et al. 2022); as it would spare patients from having to travel long distances to obtain high‐quality care (Krupinski 2015; Khoza‐Shangase and Sebothoma 2022). However, caregivers in this study demonstrated a reluctance to use this mode of service delivery. This reluctance may stem from concerns about technological literacy, connectivity issues and the perception that telehealth lacks the personal interaction required for effective care delivery. Findings of this finding are two‐fold: (1) If South Africa is to provide healthcare to all South Africans in line with the human resources for health strategy and the national health insurance bill, a substantial increase in recruitment, training and retention of audiologists is warranted especially in the public healthcare sector which caters for 84% of the population (Pillay et al. 2020); (2) careful deliberations on telehealth use within this context is essential, including a focus on awareness of and attitudes towards telehealth, internet access and use, as well as feasibility of this mode of service delivery given the country's current challenges with meeting the population's electrical demands. Exploring hybrid models that combine telehealth with occasional in‐person visits could address some of these barriers. Challenges with accessing healthcare due to distance are not unique to South Africa. Studies from rural Australia and parts of the United States highlight similar issues, underscoring the need for innovative solutions such as mobile clinics and hybrid telehealth models worldwide.

The third finding of caregivers' preference for home visits being conducted as part of early intervention is consistent with Rotheram‐Borus et al. (2017) and Tubach et al. (2012), which demonstrated that providing intervention in the family's home improves child development, caregiver involvement, caregiver participation and caregivers' sense of control and comfort. Furthermore, home visits mitigate the documented distance challenges associated with accessing EHDI services (Khoza‐Shangase 2019; Maluleke et al. 2023); and allow HCPs to offer a more tailored approach to service delivery (Peacock et al. 2013). Given the documented socio‐economic disparities, home‐based services in this context may also reduce the financial and logistical burdens on families, particularly those living in poverty. However, the feasibility of conducting home visits within the South African context requires further exploration given the logistics challenges already discussed (Ferguson 2018). Policymakers must evaluate the cost‐effectiveness of deploying trained community health workers or other innovative solutions to meet the demand for home‐based interventions.

The fourth finding of caregiver's preference for information pertaining to support services to be provided as a regular part of early intervention services highlights the importance of informational counselling for caregivers. Ninety per cent of children with hearing impairment are born to hearing parents who often have little to no knowledge of childhood hearing impairment (Birdsey and Joseph 2021; Davids and De Jager 2018). Thus, informational counselling is vital for these caregivers and has significant implications for follow‐up of treatment options, as well as commitment and adherence to treatment recommendations, and reducing the stress and burden of raising a child with hearing impairment (Maluleke et al. 2021; Turan 2012; Watermeyer et al. 2017). Caregivers require consistent, culturally sensitive counselling that addresses both the emotional and practical challenges of managing childhood hearing impairment. This information should be provided in a responsive and sensitive manner for them to be amenable to accessing follow‐up services (Khan et al. 2018; Watermeyer et al. 2017). This finding is significant for the need for informational counselling to be continuous throughout the EHDI process, while being cognizant of the family's needs, as well as the child's need and development.

Lastly, caregivers' preference for the cost of EHDI services to be decreased in an effort to make these services affordable supports findings by Naidoo and Khan (2022), and Maluleke et al. (2023) where the high cost of EHDI services were identified as a barrier to accessing these services. South Africa has one of the highest income inequalities in the world, such that the poorest 10% share about R1.1 billion compared with R381 billion shared by 10% of the richest population (Statistics South Africa 2020). This alarming maldistribution of income means that more than 84% of the population may not be able to access health services due to high costs (McIntyre et al. 2009). Post‐apartheid, the South African government aimed to improve access to healthcare by abolishing user fees for primary healthcare (Burger and Christian 2020), and this is the goal of the National Health Insurance (NHI) (World Health Organisation (WHO) 2015). However, until universal health coverage is achieved through the NHI, this finding highlights a need for restructuring the policies of addressing healthcare access needs to ensure that they are all‐inclusive in addressing the needs of all children with disabilities including children with hearing impairment. Integrating EHDI services into public health programmes and providing subsidies for hearing aids and related devices could significantly alleviate these financial burdens. Affordability remains a universal challenge in accessing healthcare services, particularly for marginalized populations in LMICs. These findings reinforce the need for global policy interventions to subsidize costs and integrate EHDI services into publicly funded health systems.

The findings of the current study must be interpreted with consideration of several limitations. First, the inclusion of caregivers from early intervention preschools located only in Gauteng limits the generalizability of the findings to the broader South African population. Gauteng, being a predominantly urban province, may not accurately reflect the preferences of caregivers from rural areas or provinces with different socio‐economic dynamics. Future studies should include a more geographically diverse sample to capture the full spectrum of caregiver preferences across South Africa. Second, the conditional logit model used in this study yielded weak correlation coefficients (*r* < 0.7) and *p* values > 0.05 for several attributes, including ‘diagnostic evaluation’, ‘aural habilitation, speech and language therapy’, ‘support services’ and ‘reducing the cost of services’. This suggests that additional, unmeasured factors may have influenced the observed relationships between attributes and attribute levels. Further qualitative research could provide deeper insights into the underlying reasons for these preferences, particularly by exploring caregivers' lived experiences and specific barriers to accessing EHDI services. While the attributes and levels included in the conjoint analysis were informed by extensive narrative interviews with caregivers, it is possible that some factors influencing caregiver preferences were not fully captured. The structured nature of conjoint analysis, which requires predefined attributes and levels, may have limited the scope for uncovering nuanced or emergent concerns that were not initially anticipated. Furthermore, caregivers' perspectives and priorities may evolve over time due to changing personal experiences, service availability and policy developments, necessitating ongoing qualitative inquiry. Future qualitative research should therefore employ a broader methodological approach, such as longitudinal qualitative studies, focus groups with diverse caregiver populations (including those in underrepresented geographical areas) and participatory research designs where caregivers actively contribute to defining key concerns. Specifically, research questions should explore decision‐making processes, trade‐offs caregivers make in accessing services and any additional factors that influence their preferences beyond those identified in this study. This approach would help uncover deeper contextual and systemic influences on caregiver choices, thereby refining the attributes considered in future preference studies.

Third, the study relied on self‐reported data collected through an online survey, which may introduce biases such as social desirability or recall bias. Additionally, the reliance on online data collection methods may have excluded caregivers without access to the internet or digital devices, which is a notable limitation given the digital divide prevalent in South Africa. Future research should consider incorporating face‐to‐face interviews or mixed methods approaches to ensure inclusivity and address potential biases. Fourth, while the sample size was limited, it aligns with previous studies utilizing discrete choice experiments in healthcare research. Nonetheless, future studies with larger and more diverse caregiver populations across multiple provinces would enhance the generalizability of the findings. Finally, the study focused exclusively on caregivers of children already enrolled in early intervention programmes. As a result, the preferences of caregivers whose children have not yet been diagnosed or who have not accessed EHDI services remain unexplored. Expanding the sample to include such caregivers could provide a more comprehensive understanding of the barriers to and facilitators of EHDI service utilization.

Despite these limitations, the study provides valuable insights into caregivers' preferences for EHDI services and highlights critical areas for intervention and policy development within the South African context.

## 5 Conclusion

This study represents a critical step towards understanding caregivers' preferences for EHDI services within the South African context. By utilizing conjoint analysis, the study highlighted key attributes that caregivers value in EHDI services, including the provision of services in their home language, diagnostic evaluations conducted at the nearest healthcare facility, home‐based early intervention services, regular and integrated support services and a reduction in the cost of EHDI services. These findings underscore the importance of developing context‐specific, family‐centred EHDI programmes that address the unique linguistic, socio‐economic and geographic challenges faced by South African families.

The preference for services provided in home languages reflects the linguistic diversity of South Africa and the critical need to ensure that communication barriers do not hinder caregivers' access to and understanding of EHDI services, as seen in other multicultural contexts. Policymakers and service providers should prioritize the recruitment and training of linguistically diverse healthcare professionals and explore the integration of interpreter services into existing programmes. Furthermore, public health campaigns should raise awareness about the benefits of home language services, fostering trust and encouraging greater engagement from caregivers. Addressing these barriers is critical for ensuring equity in EHDI services globally. The preference for diagnostic evaluations conducted at the nearest healthcare facility highlights the persistent accessibility challenges faced by many families. Innovative solutions, such as mobile clinics and hybrid telehealth models, should be explored to bridge these gaps. However, the reluctance to adopt telehealth services in this study suggests that efforts must also focus on addressing technological literacy, ensuring reliable internet access and fostering caregiver trust in virtual care delivery.

Home‐based early intervention services emerged as a key preference, underscoring the importance of tailoring services to meet families' specific needs. However, the feasibility of implementing widespread home‐based services in South Africa requires careful consideration of resource constraints. Policymakers must evaluate the cost‐effectiveness of community health worker programmes and explore partnerships with nongovernmental organizations to support this model of care. The integration of support services as a regular component of EHDI programmes highlights the critical role of informational counselling in empowering caregivers. Given that most caregivers may have limited knowledge about hearing impairment, continuous and culturally sensitive counselling should be embedded throughout the intervention process. Support services should address not only the practical aspects of managing childhood hearing loss but also the emotional and psychological challenges faced by families. This requires equipping healthcare providers with the skills and tools needed to deliver responsive and empathetic care.

Finally, the preference for reducing the cost of EHDI services emphasizes the need for affordable and equitable access to care. Policymakers should prioritize the integration of EHDI services into publicly funded healthcare programmes and advocate for subsidies or funding mechanisms to support families. Additionally, partnerships with private sector stakeholders could help reduce the cost of amplification devices and ensure that these essential tools are accessible to all who need them. To address the limitations of this study, future research should include a more geographically and socio‐economically diverse sample, incorporate mixed methods approaches and explore the preferences of caregivers who have not yet accessed EHDI services. Such studies will provide a more comprehensive understanding of caregiver needs and further refine the development of EHDI programmes tailored to the South African context.

While rooted in the South African context, this study findings offer valuable insights for global health systems seeking to improve the accessibility and equity of EHDI services. Future research should explore caregiver preferences in diverse cultural and economic contexts to identify universal strategies for optimizing EHDI programme design. By addressing linguistic barriers, enhancing service accessibility, integrating comprehensive support services and reducing financial burdens, South Africa and other LMICs can advance towards equitable and effective EHDI services that improve outcomes for children with hearing impairment and their families. Policymakers, healthcare providers and stakeholders must work collaboratively to implement these recommendations, ensuring that EHDI programmes are responsive to the diverse needs of communities.

## Author Contributions

N.P.M. contributed to conceptualization, methodology, investigation, original draft preparation, reviewing and editing. K.K.S. contributed to conceptualization, methodology, supervision, original draft preparation, reviewing and editing. All authors read and approved the final manuscript.

## Ethics Statement

The current study adhered to the Helsinki Declaration of 1975, as revised in 2013. To this end, before data collection for the study could be conducted, ethical clearance was secured from the University of the Witwatersrand's Human Research Ethics Committee (Medical) (Protocol Number: H19/06/16). To gain access to participants, written permission was obtained from two early intervention preschool centres that cater for children who are DHH in Gauteng, South Africa, allowing the researchers access to the preschool records to identify potential participants for the current study. Researchers placed posters at reception areas of the preschools delineating the study, recruitment strategy, participant inclusion and exclusion criteria, as well as methods that were involved in data collection. Written informed consent was obtained from the participants prior to data collection.

## Consent

The authors have nothing to report.

## Conflict of Interests

The authors declare no conflicts of interest.

## Data Availability

The data sets used and/or analysed during the current study are available from the corresponding author on a reasonable request.
